# 85 Achieving a sustained reduction in Staphylococcus aureus infections in the Neonatal Intensive Care Unit

**DOI:** 10.1017/ash.2026.10511

**Published:** 2026-06-23

**Authors:** Faisal Adam, Ashenafi Yirga, Cherra Hampton-Mitchell, Candace Johnson

**Affiliations:** 1 DC Health Department; 2 DC Department of Health; 3 DC Health

## Abstract

**Background:** Optimizing antimicrobial use is essential to reducing antimicrobial resistance and healthcare-associated infections. The Standardized Antimicrobial Administration Ratio (SAAR), reported through the National Healthcare Safety Network (NHSN), provides a risk-adjusted benchmark for antimicrobial utilization, yet its use as a structured feedback tool across multiple hospitals remains limited. In June 2025, a jurisdiction-led antimicrobial stewardship intervention was implemented to provide standardized, data-driven feedback to acute care hospitals. This analysis evaluates early changes in facility-level SAARs following implementation. Methods Five acute care hospitals participated in a monthly feedback intervention adapted from a previously piloted quarterly model. Public health analysts generated standardized written reports using routinely submitted NHSN SAAR data for adult inpatient antimicrobial use. Reports included facility-level SAAR benchmarking, peer comparisons across participating hospitals, and qualitative assessment of prescribing patterns, including empiric therapy selection, de-escalation opportunities, and duration of therapy. Facility-level SAAR trends from January 2024 through December 2025 were examined using descriptive time-series review. Percent change relative to the June 2025 baseline was calculated for July–December 2025. Year-over-year comparisons to the same months in 2024 were used to account for seasonality. Results Pre-intervention SAARs varied across hospitals, with several facilities demonstrating upward trajectories. Following implementation, three of five hospitals demonstrated relative declines in overall SAARs, while two hospitals showed stabilization after previously increasing trends. Percent-change analyses for July–December 2025 showed reductions of approximately 0.5% to 7% compared with the June 2025 baseline, with three hospitals achieving declines of 5–7%. Year-over-year comparisons indicated reductions of approximately 3–10% in three hospitals, with the remaining facilities showing stable use (≤2% change). Conclusions Early findings suggest that a jurisdiction-led, SAAR-based feedback model may support measurable improvements or stabilization in antimicrobial use across diverse acute care hospitals. Leveraging routinely available NHSN data to provide consistent, structured feedback represents a feasible, low-burden approach to strengthening antimicrobial stewardship efforts. Ongoing analyses will evaluate unit-specific and antimicrobial-specific SAAR categories to further characterize the intervention’s impact.

